# The Effect of Elevated Protein Intake on DNA Damage in Older People: Comparative Secondary Analysis of Two Randomized Controlled Trials

**DOI:** 10.3390/nu13103479

**Published:** 2021-09-30

**Authors:** Agnes Draxler, Bernhard Franzke, Johannes T. Cortolezis, Nicola A. Gillies, Sandra Unterberger, Rudolf Aschauer, Patrick A. Zöhrer, Laura Bragagna, Julia Kodnar, Eva-Maria Strasser, Oliver Neubauer, Pankaja Sharma, Sarah M. Mitchell, Nina Zeng, Farha Ramzan, Randall F. D’Souza, Scott O. Knowles, Nicole C. Roy, Anders M. Sjödin, Cameron J. Mitchell, Amber M. Milan, Barbara Wessner, David Cameron-Smith, Karl-Heinz Wagner

**Affiliations:** 1 Department of Nutritional Sciences, University of Vienna, 1090 Vienna, Austria; agnes.draxler@univie.ac.at (A.D.); bernhard.franzke@univie.ac.at (B.F.); johannes.theodor.cortolezis@univie.ac.at (J.T.C.); patrick.zoehrer@univie.ac.at (P.A.Z.); laura.bragagna@univie.ac.at (L.B.); Julia.kodnar@univie.ac.at (J.K.); 2 Research Platform Active Ageing, University of Vienna, 1090 Vienna, Austria; sandra.unterberger@univie.ac.at (S.U.); rudolf.aschauer@univie.ac.at (R.A.); oliver.neubauer@univie.ac.at (O.N.); barbara.wessner@univie.ac.at (B.W.); 3 Liggins Institute, University of Auckland, Auckland 1142, New Zealand; n.gillies@auckland.ac.nz (N.A.G.); p.sharma@auckland.ac.nz (P.S.); sarah.mitchell@auckland.ac.nz (S.M.M.); n.zeng@auckland.ac.nz (N.Z.); f.ramzan@auckland.ac.nz (F.R.); r.dsouza@auckland.ac.nz (R.F.D.); nicole.roy@otago.ac.nz (N.C.R.); cameron.mitchell@ubc.ca (C.J.M.); a.milan@auckland.ac.nz (A.M.M.); DCameron_Smith@sics.a-star.edu.sg (D.C.-S.); 4 Riddet Institute, Massey University, Palmerston North 4442, New Zealand; 5 Centre for Sport Science and University Sports, University of Vienna, 1150 Vienna, Austria; 6 Karl Landsteiner Institute for Remobilization and Functional Health/Institute for Physical Medicine and Rehabilitation, Kaiser Franz Joseph Hospital, Social Medical Center South, 1100 Vienna, Austria; eva-maria.strasser@wienkav.at; 7 Center for Health Sciences and Medicine, Danube University Krems, 3500 Krems, Austria; 8 Discipline of Nutrition, Faculty of Medical and Health Sciences, University of Auckland, Auckland 1142, New Zealand; 9 Maurice Wilkins Centre for Molecular Biodiscovery, University of Auckland, Auckland 1142, New Zealand; 10 Smart Foods Innovation Centre of Excellence, AgResearch, Palmerston North 4410, New Zealand; scott.knowles@agresearch.co.nz; 11 Department of Nutrition, University of Otago, Dunedin 9054, New Zealand; 12 Department of Nutrition, Exercise, and Sports, Copenhagen University, 2200 Copenhagen, Denmark; amsj@nexs.ku.dk; 13 School of Kinesiology, University of British Columbia, Vancouver, BC V6T 1Z4, Canada; 14 Singapore Institute for Clinical Sciences, Agency for Science, Technology and Research, Singapore 138632, Singapore

**Keywords:** protein intake, elderly, DNA damage, comet assay, glutathione

## Abstract

A high protein intake at old age is important for muscle protein synthesis, however, this could also trigger protein oxidation with the potential risk for DNA damage. The aim of this study was to investigate whether an increased protein intake at recommended level or well above would affect DNA damage or change levels of reduced (GSH) and oxidised glutathione (GSSG) in community-dwelling elderly subjects. These analyses were performed in two randomized intervention studies, in Austria and in New Zealand. In both randomized control trials, the mean protein intake was increased with whole foods, in the New Zealand study (*n* = 29 males, 74.2 ± 3.6 years) to 1.7 g/kg body weight/d (10 weeks intervention; *p* < 0.001)) in the Austrian study (*n* = 119 males and females, 72.9 ± 4.8 years) to 1.54 g/kg body weight/d (6 weeks intervention; *p* < 0.001)). In both studies, single and double strand breaks and as formamidopyrimidine—DNA glycosylase-sensitive sites were investigated in peripheral blood mononuclear cells or whole blood. Further, resistance to H_2_O_2_ induced DNA damage, GSH, GSSG and CRP were measured. Increased dietary protein intake did not impact on DNA damage markers and GSH/GSSG levels. A seasonal-based time effect (*p* < 0.05), which led to a decrease in DNA damage and GSH was observed in the Austrian study. Therefore, increasing the protein intake to more than 20% of the total energy intake in community-dwelling seniors in Austria and New Zealand did not increase measures of DNA damage, change glutathione status or elevate plasma CRP.

## 1. Introduction

The age-related loss of muscle mass, function and strength, well known as sarcopenia or dynapenia, decreases mobility and the ability to perform daily activities in elderly people [1,2]. In order to maintain muscle mass and physical function until old age, dietary nutrient intake is critical and in particular protein is most essential for muscle protein synthesis. Official dietary protein recommendations for older people differ widely worldwide and are usually in the range between 0.8 and 1.2 g/kg body weight (BW)/d [3]. However, there is emerging evidence that in healthy older adults a higher protein intake could be beneficial, particularly for muscle metabolism [4,5]. Further, various societies related to aging, sarcopenia, gerontology or clinical nutrition have made recent recommendations for a daily protein intake in the range of 1.0–1.5 g/kg BW to optimally support health (e.g., [6,7,8]).

However, any recommendation for dietary change in the elderly must consider all aspects of health, not just muscle mass. The importance of accumulating free radical damage, increased oxidative stress markers, diminished anti-oxidant defenses are all well described as being key components of the ageing process. This includes heightened DNA damage. Collectively, the increased oxidative stress and damage increases the susceptibility for the many complex diseases that are experienced by the elderly [9].

There have been few studies that have addressed the possible impact that higher protein intake may exert on protein oxidation. This is an important consideration as protein oxidation is critically important for cellular processes, which are redox-regulated mainly by thiol-oxidation [10]. Protein oxidation can therefore result in sustained damage of a protein structure, with the possible loss in function.

That an interaction of the DNA with proteins to protect or induce DNA damage is possible was already shown in a computational study [11]. Van-Hecke et al., [12] found recently an increase of lipid oxidation and protein carbonylation during digestion of red meat and demonstrated the formation of a genotoxic DNA adduct in a simulated colonic environment. The same group [13,14] confirmed that severe lipid and protein oxidation occurred concomitantly with an intense formation of O6-carboxy-methylguanine in a simulated colonic environment, strongly implicating not only lipid—but also protein oxidation products in the DNA damage that would initiate the carcinogenic process.

Following a single meal, the ingestion of protein (casein) triggers a burst of reactive oxygen species (ROS) generation from circulating white blood cells, although it must be noted that this is less than what may be experienced with a high fat meal [15]. Yet this theoretically suggests the possibility for heightened oxidative stress to high protein meals. Experimentally, Petzke et al. (2000) did not demonstrate increased oxidative stress after feeding up to 51 E% of protein to adult rats for 15 weeks [16]. In the very limited clinical analysis, Pivovarova-Ramich et al. (2020) investigated the effects of diets high in animal or plant-based protein sources on oxidative stress markers in older patients with type 2 diabetes. Six weeks of 30 E% given as plant-/animal-based protein decreased malondialdehyde and protein carbonyls, but increased nitrotyrosine [17].

In some studies, protein was administered together with a resistance training program, yet few have investigated measures of oxidative stress. With twelve weeks of whey protein supplementation (3× week, 35 g pre and post training) Nabuco et al. (2019) demonstrated reduced plasma uric acid concentrations but did not further effect antioxidant enzyme activity or oxidative stress markers in older women [18]. Similar a 12 week exercise and protein supplementation study (to achieve 1.5 g protein/kg BW/d) in older Malays lead only to a reduction in superoxide dismutase levels in the exercise but not the protein group and did neither alter protein nor lipid oxidation markers [19].

There are no data so far investigating potential effects of a high protein diet, on measures of oxidative stress and DNA damage in older adults. We have recently shown that 6-month progressive resistance training, with or without protein and vitamin supplementation in 105 Austrian institutionalized women and men (65–98 years), increased basal DNA damage in both groups after 3 and 6 months of intervention [20]. However, this study emphasized an increased protein intake via supplements.

To better understand the impact of a high protein intake using whole foods on oxidative damage to the DNA, the aim of this investigation was to undertake a secondary analysis of two complementary clinical trials. We used data from two randomized control trials with a similar study background, one applied in Vienna (further referred to as the Austrian study) and the other performed in Auckland (further referred to as the New Zealand study) and considered them in this paper. In both studies the protein intake in community dwelling older adults was increased in a similar range and single and double strand breaks were investigated in peripheral blood mononuclear cells (PBMC) or whole blood as well as formamidopyrimidine—DNA glycosylase (FPG)-sensitive sites. Further, resistance to H_2_O_2_ induced DNA damage and reduced (GSH) and oxidized (GSSG) glutathione were measured as GSH is known to contribute to the inhibition of DNA damage and plays a central role in eliminating peroxides [21].

## 2. Materials and Methods

### 2.1. Austrian Study

#### 2.1.1. Experimental Design Austrian Study

The study was designed as a randomized controlled, observer-blind trial and was part of the larger NutriAging study, which consisted of two intervention periods, a nutritional intervention for six weeks followed by a nutritional and strength training intervention of further eight weeks. The study was conducted from July to December 2018. Inclusion and exclusion criteria were screened before the beginning of the study. In order to better compare the Austrian to the New Zealand study, we only used data from the first six weeks of nutritional intervention with a different protein load. Data were collected before and after the six weeks nutritional intervention.

#### 2.1.2. Participants Austrian Study

A total of 632 people expressed interest in the study, with 183 undergoing a medical screening. Of these 136 met the eligibility criteria and were recruited into the study. Two participants withdrew due to medical reasons shortly after randomization, therefore baseline data are available for 134 individuals. From baseline (‘Pre-’) to the end of the protein intervention (‘Post-’) 15 people dropped out of the study so 119 participants completed the protein intervention. Inclusion criteria included community-dwelling men and women aged between 65 and 85 years who did not perform any regular resistance training during the last six months prior to the study. Cognitive impairment (Mini Mental State Examination Score < 23), acute and chronic diseases that would contraindicate the training sessions, serious cardiovascular diseases, diabetic retinopathy, manifest osteoporosis, anticoagulant or cortisone medication, a frailty index ≥ 3 or the need for walking aids comprised the exclusion criteria. Pre-existing diseases such as hyperlipidaemia, diabetes mellitus type 2, osteoporosis, heart disease and history of cancer were evaluated during the medical examination. Obesity (BMI ≥ 30 kg/m^2^) and hypertension (blood pressure above 140/90 mm Hg) were defined according to World Health Organization (WHO) criteria [22]. A written informed consent form was signed by all participants before participation in the study. The study was performed in accordance with the Declaration of Helsinki, approved by the Ethics Committee of the University of Vienna (Reference Number: 00322) and registered at https://clinicaltrials.gov (NCT04023513, 17 July 2019).

#### 2.1.3. Nutritional Intervention Austrian Study

Participants were allocated to control (CON), recommended protein (RP) or the high protein (HP) groups. The food-based interventions were personalized and isocaloric. For RP a target protein intake of 1.0 g/kg BW/d should be achieved based on the recommendation of the D-A-CH reference values, which are valid for the German speaking countries in Europe [23]. For HP the target protein intake should be the doubled recommendation, and while this could not be achieved during the six-week period, we were able to almost double their habitual protein intake, which was approximately 0.8 g/kg BW/d, to achieve 1.54 g/kg BW/d. The additional protein intake in the HP group was not accomplished with protein supplements, but via nutritional counselling and protein-rich whole foods (e.g., protein rich milk products, bars, puddings, protein rich bread, bacon crisps, protein rich soups, pea protein sticks as well as recipes for self-prepared foods). The RP group also received similar foods with moderate protein content (e.g., milk products, bars, bread, soups, self-made vegetable muffins) to be isocaloric. The protein intake was personalized and calculated on the respective body weight and monitored over the entire study. The subjects received a fortnightly delivery of the food for the calculated additional protein intake via special delivery service. For the CON, the subject’s habitual dietary intake was monitored but not changed during the whole study period. The sex specific target range for total energy intake for HP and RP was based on the D-A-CH reference values for this age group for physical activity (PAL) levels of 1.4 to 1.6 (females: 1700–1900 kcal/d; males: 2100–2500 kcal/d). Physical activity was assessed with the German-PAQ-50+ questionnaire [24].

#### 2.1.4. Dietary Intake Assessment Austrian Study

The participants had to complete a 24-h dietary recall periodically (every 7–10 days) over the study period to record the habitual as well as the additional protein rich food intake accurately. 5 ± 1 interviews per participant were performed and evaluated within 2 days on average. For the pre-intervention food intake two 24-h dietary recalls were performed within 10 days. Since 24-h recalls were conducted on non-consecutive days, four out of five interviews were carried out based on a weekday and one based on a weekend day. Dietary intake was assessed by using Globodiet^®^, formerly EPICSoft. GloboDiet was developed by the International Agency for Research on Cancer and further adapted for Austria at the Department of Nutritional Sciences in Vienna. Every participant received a photo book to support the estimation of the portion size. The reported foods collected during the interviews were linked to the German food composition database (Bundeslebensmittelschlüssel) version 3.02. Total energy intake (kcal), carbohydrates (g/kg BW/d), fat (g/kg BW/d) and protein (g/kg BW/d) were estimated. Furthermore, participants recorded the consumption of all provided products on a daily basis in a food diary. These logs were collected and reviewed during every 24-h recall. They also received nutritional training, especially regarding the amount of protein of the different products they received and they additionally received a study book, which also contained this information. The information on the protein intake of each participant was evaluated within 2 days after the respective interview to adjust for the protein intake when needed.

### 2.2. New Zealand Study

#### 2.2.1. Experimental Design New Zealand Study

The design of the OptiMuM study (Optimal nutrition in the elderly: High protein diets for muscular, metabolic, and microbiome health) has been published in detail [25,26]. This was a parallel-group design in which elder male participants were allocated (1:1) to consume either the RDA (0.8 g/kg BW/d) or twice the RDA (2RDA, 1.6 g/kg BW/d) of protein for 10 weeks. All participants provided informed written consent prior to enrollment in the trial. The study was prospectively registered with the Australian and New Zealand Clinical Trial Registry (www.anzctr.org.au, 9 March 2016) as ACTRN12616000310460, approved by the Southern Health and Disability Ethics Committee (Ministry of Health, Wellington, New Zealand; 15/STH/236), and conducted in accordance with the Declaration of Helsinki. The primary outcome of the OptiMuM study was to evaluate changes in skeletal muscle mass and function in healthy older men [25]. The secondary outcomes reported in this article include assessment of DNA damage.

#### 2.2.2. Participants New Zealand Study

Thirty-one healthy older men (>70 y) living in Auckland, New Zealand were recruited through advertisements in local newspapers. Eligible participants included those with a body mass index between 18 and 35 kg/m^2^, non-smokers, and could perform activities of daily living independently without mobility aids. Ineligibility criteria included prior history of major diseases (including cancers, diabetes, and thyroid diseases), regularly dietary supplements use and unwillingness to abstain for at least 1 month before and during the trial, or those with restrictive eating habits (e.g., vegetarians and those with intolerances and allergies). Further exclusion criteria included individuals with conditions affecting skeletal neuromuscular function or those performing over 4 h per week of structured physical activity (organized sport, resistance training, or vigorous-intensity aerobic exercise). All testing was conducted between April and October 2016 at the Liggins Institute (University of Auckland).

#### 2.2.3. Nutritional Intervention New Zealand Study

Participants had all food during the 10-week intervention period delivered to their homes, including breakfasts, lunches (Muscle Chow NZ Ltd., Auckland, New Zealand), evening meals (Farmhouse Foods Ltd., Auckland, New Zealand), plus additional snacks. In order to maintain adherence, all items were portioned and required only minimal preparation or reheating. Participants were able to self-select from a range of meal options, with alterations made to suit individual preferences where feasible. Participants were able to drink water, tea, and coffee without restrictions, and were instructed to maintain their normal lifestyle.

The energy content of each participant’s intervention diet was individually calculated to match the participant’s estimated energy requirements, which was adjusted fortnightly int eh study based on physical activity level and self-reported satiety. Individual energy estimates were based on the Harris–Benedict equation with adjustment physical activity level, which was measured by wrist-worn accelerometers (Fitbit Charge HR, San Francisco, CA, USA). Accelerometers were worn for 5-day periods prior to the intervention, and at weeks 5 and 10.

Dietary protein was from a variety of sources, including plant-derived (whole grains, legumes, nuts, dairy products), white meat (chicken, fish), and red meat (beef) for both RDA and 2RDA diets. Smaller portions of animal-based proteins were provided in the RDA diet, while the ratio of animal protein sources to plant protein sources was higher in the 2RDA diet [25]. Both treatment groups adhered to the New Zealand Eating and Activity Guidelines [27], meeting minimum recommendations for major food groups (fruits and vegetables, grains, dairy products/alternatives, meat/alternatives). Adherence records were checked at week 5, 6, 9, and 10, where participants indicated both the proportion of each provided meal consumed and reported any food consumed not provided.

#### 2.2.4. Dietary Intake Assessment New Zealand Study

Participants completed a 3-d food record before the intervention began to estimate habitual intake. Follow-up dietary intake was estimated using their 7-day personalized meal plans from week 10 of the intervention. Food and beverage intake was analyzed by a registered dietitian using FoodWorks (Version 9; Xyris, Brisbane, Australia), which aligns with the New Zealand Food Composition Database (New Zealand FOODfiles 2016, Version 01).

### 2.3. Assessment of DNA Damage

Oxidative damage to DNA was measured in Vienna for both studies with the same method, the single cell gel electrophoresis (comet assay) as described previously [20,28]. The comet assay for PBMC for the NZ samples was performed according to the method described by Azqueta et al. [29] in a 12-minigel format [30]. The comet assay for whole blood for the Austrian study was performed according to Al-Salmani et al. [31] with slight modifications. Stored whole blood was thawed quickly, 10 μL whole blood was mixed with 200 μL 1%-agarose solution and 5 μL were pipetted on the respective spot of the 12-minigel slide. The same procedure was applied to thawed and washed PBMC samples (which were prepared in New Zealand and sent to Austria on dry ice), whereby 15 μL cell solution with a concentration of 5 × 10^5^ cells/mL (in phosphate buffered saline solution) was mixed with 70 μL 1%-agarose solution and 5 μL were pipetted on the respective spot of the 12-minigel slide. Each sample was analyzed in duplicates, resulting in 6 different samples per 12-minigel slide. For each 6-sample-group, 4 slides were necessary for the following treatments: lysis, buffer, formamidopyrimidine—DNA glycosylase (FPG) and H_2_O_2_ for PBMC and whole blood respectively. All following steps were done equally for whole blood and PBMC slides.

Slides were placed in lysis solution for one hour. H_2_O_2_ slides were treated with a 100 μM H_2_O_2_ solution for 5–15 min. at 4 °C prior to lysis. After lysis, FPG and buffer slides were washed three times with cold enzyme buffer (40 mM HEPES, 0.1 M KCL, 0.5 mM EDTA, 0.2 mg/mL BSA, pH 8) before being clamped into slide units (12-Gel Comet Assay Unit™, Severn Biotech Limited, Kidderminster, UK). The units were placed on ice and gels were respectively treated with either 30 μL enzyme buffer or 30 μL FPG solution (1:3000/3500 dilution; FPG, New England Biolabs GmbH) for 30 min at 37 °C. Thereafter, all slides were put in cold electrophoresis solution (0.3 M NaOH, 1 mM EDTA, pH > 13) for 20 min unwinding phase, followed by 30 min of electrophoresis (25 V, 300 mA at 4 °C). Slides were then washed with phosphate buffered saline solution and distilled water. For drying of the gels, slides were first placed in 70% ethanol and second in ethanol absolute for 15 min respectively. Gels were stained with GelRed (PAGE GelRed Nucleic Acid Gel Stain, Biotium). DNA damage was expressed in % Tail DNA which was quantified using a fluorescence microscope (Nikon) and the imaging software Comet Assay IV (Perceptive Instruments Ltd.). For each sample 100 cells were scored (50 per duplicate) and means were calculated. FPG-sensitive sites were expressed as net % Tail DNA.

### 2.4. Assessment of Oxidised and Reduced Glutathione

GSSG and GSH were analyzed with use of N-Ethylmaleimide and O-phthalaldehyde according to an adopted method of Hissin and Hilf [32] as described previously [33]. All samples were analyzed fluorometrically in triplicates with external standards of GSSG and GSH using a BMG FLUOstar OPTIMA Microplate Reader (BMG LABTECH GmbH, Ortenberg, Germany).

### 2.5. Anthropometry

Anthropometric measures consisted of height and weight measurements, as well as the subsequent calculation of the subjects’ body mass index (BMI). Weight and height were evaluated with a medical scale (Austrian study: model 877, seca GmbH & Co. KG, Hamburg, Germany; NZ study: Tanita DH-351) and a stadiometer. Body mass index (BMI) was calculated as weight in kg divided by height in m squared. In the Austrian study, also waist circumference (WC) and hip circumference (HC) were measured by tape (model 203, Seca) and the waist-to-hip ratio (WHR) was calculated.

### 2.6. Statistics

#### Statistical Analyses for Both Studies

The evaluation was based on an intention-to-treat analysis. For data clearance, all entries to the statistical software (IBM SPSS Statistics 26.0) were controlled twice by two independent researchers.

Normal distribution of metric variables was tested by visual inspection of the histograms as well as by Shapiro Wilk test. An appropriate transformation for all affected parameter was performed, if the normal distribution was violated.

Differences between groups at baseline were tested by one-way ANOVA or *t*-test for metric variables or chi square test for categorical measures. For assessment of time (within subject factor), group (between subject factor) and time x group interaction effects, a two-way mixed ANOVA with Bonferroni-corrected post hoc analyses was used. Homogeneity of variances was tested either by Levene-test and/or by Mauchly’s test of sphericity. If the latter was violated, Greenhouse Geisser-corrected values are shown. Simple main effects for group and time were analyzed in the case of a significant group x time interaction. Non-significant interactions were interpreted by the main effects for time and group. In order to compare the rate of changes between pre and post, differences were calculated (post-pre) and further examined by paired sampled *t*-tests. Linear correlations were calculated using the Spearman test. Significance was set to α = 0.05.

## 3. Results

### 3.1. Baseline Characteristics and Nutrient Intake

#### 3.1.1. Baseline Characteristics and Nutrient Intake Austrian Study

In total 136 older men and women were randomly allocated to CON (*n* = 47), RP (*n* = 41) and HP (*n* = 48). Two participants withdrew shortly after randomization therefore, baseline data are available for 134 individuals. 119 participants finished the entire six-week protein intervention, CON (*n* = 41), RP (*n* = 37), and HP (*n* = 41).

At baseline, dietary intake data are available from 124 subjects, as nine people left the study between baseline and their first 24-h recall and one person had no recorded data.

Groups were comparable regarding age, anthropometric measurements and body composition (Table 1). Total energy and macronutrient intake did not differ significantly between groups at baseline. Average protein intake was 0.85 g/kg/BW (14 E%) across all participants. Baseline energy-scores as assessed by the German PAQ-50+ questionnaire was 8211 ± 4830 kcal/wk in CON, 11,106 ± 8077 kcal/wk in RP and 10,065 ± 5398 kcal/wk in HP (*p* = 0.096) and did not change during the intervention.

Further, for all plasma lipid parameter, marker for DNA damage as well as oxidative stress there were no difference at baseline between groups. Only for C-reactive protein (CRP) a significant group effect was observed (*p* = 0.013).

#### 3.1.2. Baseline Characteristics and Nutrient Intake New Zealand Study

In total, 29 older men (age, 74.2 ± 3.6 y) completed the 10 week dietary intervention and were included in final analysis, as previously described [25]. One participant withdrew before the intervention (RDA group), and one was excluded from follow-up due to non-adherence (2RDA group). No differences in participant characteristics at baseline were observed between intervention groups except for total fat intake (g/d), which was higher in the RDA group (Table 2).

The number of steps taken per day before the start of the intervention was 7879 ± 3106 and 8740 ± 2699 in RDA and 2RDA groups, respectively (*p* = 0.641) and did not change during the intervention (*p* = 0.593) [25].

### 3.2. Macronutrient and Total Energy Intake

#### 3.2.1. Macronutrient and Total Energy Intake Austrian Study

As shown in Table 3, significant effects of time, group and time*group interactions were found for protein (*p* < 0.001). Average protein intake was increased by 0.19 and 0.75 g/kg BW/d (*p* = 0.002; *p* < 0.001) between pre and post in RP (pre: 14 E%, post: 15 E%) and HP (pre: 13 E%, post: 23 E%), respectively, while CON remained unchanged (pre: 13 E%, post: 13 E%). Post intervention HP had higher protein intake levels in comparison to both, RP and CON (*p* < 0.001).

No time*group interaction was found for total energy, carbohydrate and fat intakes. Energy intake increased significantly within the study period by 175 kcal/d from pre to post (*p* = 0.003). There were no differences in energy intake between groups. No differences between groups or changes over time were observed for carbohydrate or fat intake (Table 3).

#### 3.2.2. Macronutrient and Total Energy Intake New Zealand Study

As shown in Table 4, significant effects of group and time*group interactions were found for protein (*p* < 0.001). Average protein intake was increased by 0.54 g/kg BW/d (*p* < 0.001; pre: 19 E%, post: 20.2 E%) between pre and post in the 2RDA group and decreased by 0.43 g/kg BW/d (*p* <0.001; pre: 17.7 E%, post: 11.6 E%) in the RDA group. After the intervention the 2RDA group had higher protein intake levels in comparison to the RDA group (*p* < 0.001).

No time*group interaction was found for carbohydrate and fat intake. Carbohydrates increased in both groups (time effect *p* < 0.001). For the energy intake there was an increase only in the 2RDA group (*p* < 0.001; Table 4).

### 3.3. Baseline Correlations between Markers of DNA Damage and Biochemical Parameter in the Austrian Study

We observed numerous correlations between markers of oxidative DNA damage and lipid metabolism (Table 5), which were even stronger in women than in men (Supplementary Table S1). No correlations were found between DNA damage markers and anthropometric parameters. For GSH and GSSG some associations (e.g., body mass; r = 0.264, *p* < 0.05; WC: r = 0.278, *p* < 0.05; HDL-C r = −0.214, *p* < 0.05) were observed, but they were not strong or consistent.

In the New Zealand study no baseline correlations were found, which was assumingly mainly due to the lower number of subjects enrolled in this study.

### 3.4. Intervention Effects

There was neither a group effect nor a time*group interaction regarding changes in markers for oxidative DNA damage, GSH/GSSG or CRP in both studies (Table 6 and Table 7). In the NZ study there was no time effect, but in the Austrian study we could observe a small but significant time effect for all groups towards a reduction for %DNA in tail after the treatment with lysis buffer (*p* = 0.002), H_2_O_2_ challenge (*p* < 0.001) and FPG (*p* < 0.001) after 6 weeks of intervention. The same was true for reduced GSH (*p* = 0.002), which was also reduced over time for all groups. For all other parameters neither time or group effects nor a time*group interaction were assessed. The sex specific results are shown in Supplementary Tables S2 and S3.

After six weeks of intervention some changes (Δ) of DNA-damage markers particularly with plasma lipid parameters but also the WHR were observed in the Austrian study:

RP group:

Δ Lysis is correlating with Δ plasma: total-C (r = 0.409, *p* < 0.05), LDL-C (r = 0.523, *p* < 0.05), HDL-C (r = −0.355, *p* < 0.05), TG (r = 0.764, *p* < 0.01), TG:HDL-ratio (r = 0.817, *p* < 0.01); Δ WHR (r = 353, *p* < 0.05).

Δ H_2_O_2_ is correlating with Δ plasma: total-C (r = 0.419, *p* < 0.01), LDL-C (r = 0.417, *p* < 0.05), TG (r = 0.448, *p* < 0.01), TG:HDL-ratio (r = 0.473, *p* < 0.01) as well as the Δ WHR (r = 0.332, *p* < 0.05).

HP group:

Δ FPG is correlating with Δ plasma LDL-C (r = 0.327, *p* < 0.05).

CON group:

Δ Lysis is correlating with Δ plasma: HDL-C (r = −0.325, *p* < 0.05), TG (r = 0.454, *p* < 0.01), TG:HDL-ratio (r = 0.462, *p* < 0.01) and Δ WHR (r = −0.375, *p* < 0.05).

## 4. Discussion

The main aim of the present paper was to investigate whether an increase in protein intake to or well above the recommended level would affect damage to the DNA or change levels of reduced and oxidised glutathione in community-dwelling elderly subjects in two independent studies. We were able to include data of two randomized control trials with very similar study aims. In the Austrian study 119 participants (54%/46% females/males) finished the 6 week intervention. The participants of the Austrian study were able to reach the D-A-CH recommended daily level of 1.0 g protein per kg BW/d in the RP, whereas the high protein group doubled their baseline intake levels to almost 1.6 g protein per kg BW/d. In the New Zealand study 29 males finished the 10 week intervention consuming either 0.8 g protein per kg BW/d (RDA in New Zealand) or 1.6 g protein per kg BW/d (2RDA). High protein intake in both studies increased to 1.5–1.7 g protein per kg BW, whereas the RDA group (NZ) and the control group (Austria) remained at around 0.8–0.9 g protein per kg BW/d. In Austria, a third group was included which was set to the protein recommendations for elderly people in the German speaking countries which are 1 g protein per kg BW/d. It is important to note, that all analytical assessments regarding DNA damage and glutathione were performed in the same laboratory. None of the interventions led to group effects regarding DNA damage markers, GSH/GSSG levels or CRP. Therefore, increasing the protein intake to more than 20% of the total energy intake in community-dwelling seniors (average age 72.9 ± 4.8 and 74.2 ± 3.6 in Austria and New Zealand, respectively) did not increase measures of DNA damage, alter GSH, GSSG levels or its ratio or lead to an increase in CRP.

Prior to the dietary interventions, slightly higher DNA damage was evident in female participants, compared to males, for the Austrian study. This was however only significant for the H_2_O_2_ challenge (males: 10.25 ± 1.93% DNA in tail; females 11.02 ± 2.38%DNA in tail; *p* = 0.004). The New Zealand study was performed in males only. The findings in the Austrian study appear to be different to that reported previously in younger individuals, where it was shown that men tend to have a higher rate of DNA damage than women [34,35,36], however, data in elderly people are rare. At younger age females usually show a better lifestyle and higher level of oestrogen, which provides them with a better defence system against free radical species [37]. This is diminished in elderly postmenopausal women and the advantage of women gradually disappears after menopause, which leads e.g., to a higher risk of cardiovascular disease in postmenopausal women than men of the same age, a trend that is largely attributed to the role of female oestrogen [38]. This would also explain why sex specific differences in DNA damage seen at a younger age is no longer present in elderly women.

The presented studies differ to previous high protein randomized control studies, since most of them were performed by modulating the protein intake via supplements [39], not with whole foods. In the Austrian and New Zealand studies protein intake was personalised and the required food supply was calculated according to the respective body weight, which was particularly challenging for subjects of high body weight.

In the Austrian study subjects in the HP group increased their mean protein intake from 13 E% to 23 E% within 6 weeks, which was almost a doubling of their habitual protein intake (before: 0.79 ± 0.31 vs. after: 1.54 ± 0.31 g protein/kg BW/d, *p* < 0.001). In the RP (after: 1.08 ± 0.32 g protein/kg BW/d) we achieved the recommended level and the CON remained stable in their protein intake (after: 0.91 ± 0.36 g protein/kg BW/d). The other macronutrients did not change and also for energy intake we did not observe a group or a time*group effect.

In the New Zealand study the habitual intake of 1.1–1.2 g protein/kg BW/d had to be reduced in the RDA group to achieve 0.9 ± 0.1 g protein/kg BW/d (*p* < 0.001), but was increased in the 2RDA group to 1.7 ± 0.1 g protein/kg BW/d (*p* < 0.001). Both groups were slightly energy deficient, with no difference between groups (*p* = 0.427).

DNA damage as well as GSH and GSSG levels were not affected by the intervention and did not increase based on the high protein diet, in neither of the two studies. Therefore, increasing the protein intake with food sources in older males and females for 6 and 10 weeks respectively did not affect pathways, which may trigger an increase in DNA strand breaks in PBMC and in whole blood. Since this is the first study in elderly investigating this link, we can only compare data to a similar study in institutionalised subjects, which received a multivitamin and macronutrient containing supplement [20]. Here a significant increase in %DNA in tail was observed after 3 and 6 months of intervention, however, in this study the group which received the supplement performed also resistance training for the same duration which might have been responsible for this increase. The latter is supported since another group in this study, which performed only resistance training without the supplement also increased DNA damage in the same range.

In the Austrian study further interesting results were obtained. We observed a time effect for all groups with reductions in GSH and %DNA in tail, independent of whether cells were challenged with or without H_2_O_2_ and FPG. From the biological point of view the effects were rather small, yet statistically significant. This might be explained by a seasonal variation. It is already known that seasonal variations such as air temperature, sun radiation and sun insolation are predictive for the comet assay [40]. The factors contributing to a greater DNA damage in summer are longer sun and heat exposure, use of sunbeds and higher sun radiation [41]. Very recently it was shown in adults that greater baseline DNA damage was observed in summer showing the highest levels of DNA damage compared with all the other seasons [40]. This would also explain the time effect in the Austrian study part, since we started with the investigations mid of July to end of August 2018. During this time period heat waves were experienced, with 29 out of 48 days during the recruitment period experiencing daily peak temperatures above 30 °C, with 45 days out of 48 exceeding 25 °C. In September and October, when the second investigation was performed, only 3 days were above 30 °C and 16 above 25 °C.

In the New Zealand study which was performed between April and October the temperature was more stable with no single day above 25 °C within the study months [42]. Therefore, this seasonal effect and the annual changes in the weather depend on the location where studies are being performed and should be taken into consideration in the future when planning cohort or intervention studies, particularly in times of a changing climate.

Further some interesting and strong correlations of DNA damage marker with lipid metabolism were found both at baseline and after intervention. Interestingly, body composition or physical performance was not associated with baseline DNA damage, although we could recently demonstrate that higher aerobic fitness significantly correlates with a lower rate of chromosomal damage [43].

The studies have several strengths and limitations. First, this manuscript combines two randomized intervention trials, independently conducted and both using a food-based approach to increase the protein intake in elderly subjects. This is rare compared to the use of protein supplementation, particularly in this age group. Further, the dietary intake was personalised, strictly controlled in both studies and modulated as and when required. Although the target dietary protein intake level of 2 g/kg BW/d in the Austrian study was not reached, the mean 1.54 g protein/kg BW/d was almost double baseline intake. The analyses of DNA damage marker and glutathione assessment were performed in the same lab in Austria, so lab-based differences, which are well known for the comet assay [44] could be excluded. The start of the Austrian study was in summer 2018, which consisted of many days above 30 °C. This might be the reason for observing the time effect for all groups regarding DNA damage; a fact that must be taken into account in future studies.

## 5. Conclusions

Two randomized control trials could clearly show that a substantial increase in the habitual protein intake to 1.5–1.7 g/kg BW/d in elderly community-dwelling subjects with a whole food approach is possible. This dietary modification did not lead to changes in single and double strand breaks, neither in the standard comet assay nor after the use of the restriction enzyme FPG or a H_2_O_2_ challenge, and levels of oxidized and reduced glutathione. Comparisons with literature data were challenging, since only very few studies have been published with a similar background, so this situation should encourage to further investigate the link between protein intake or other nutrient related changes in elderly subjects and DNA damage and oxidative stress parameter. However, on the basis of the presented intervention trials, in the period of analysis, there was no marked dietary effect on measures of oxidative stress and DNA damage. Importantly, when conducting human intervention trials over months and considering the comet assay as outcome marker, the seasonal effects should be taken into consideration when planning a trial.

## Figures and Tables

**Table 1:** Baseline characteristics and nutrient intake of the participants in the Austrian study.

	Total	CON	RP	HP	*p*-Value
Sex [f/m], (% females)]	73/63 (54.4%)	24/23 (53.2%)	21/20 (51.2%)	28/20 (58.3%)	0.724
Age [years]	72.9 ± 4.8	73.0 ± 4.9	72.4 ± 4.3	73.2 ± 5.2	0.732
Body mass [kg]	74.3 ± 13.6	74.3 ± 13.0	75.3 ± 15.3	73.4 ± 12.7	0.808
Height [m]	1.68 ± 0.1	1.69 ± 0.1	1.69 ± 0.1	1.67 ± 0.1	0.640
BMI [kg/m^2^]	26.2 ± 3.9	26.0 ± 4.0	26.3 ± 4.3	26.2 ± 3.6	0.938
Energy intake [kcal/d]	1845 ± 714	1838 ± 726	2000 ± 693	1719 ± 710	0.205
Protein intake [g/kg BW/d]	0.85 ± 0.43	0.83 ± 0.40	0.97 ± 0.55	0.78 ± 0.33	0.129
Protein intake [g/d]	62.3 ± 29.6	60.3 ± 27.8	71.6 ± 34.7	56.2 ± 24.8	0.052
Fat intake [g/kg BW/d]	1.07 ± 0.51	1.08 ± 0.56	1.12 ± 0.45	1.02 ± 0.53	0.681
Fat intake [g/d]	78.2 ± 39.1	78.2 ± 40.0	83.6 ± 37.5	73.8 ± 39.8	0.530
Carbohydrate intake [g/kg BW/d]	2.61 ± 1.24	2.68 ± 1.38	2.76 ± 1.09	2.43 ± 1.22	0.431
Carbohydrate intake [g/d]	188.5 ± 83.7	192.7 ± 89.0	204.9 ± 83.7	170.9 ± 76.9	0.171
Lysis [%DNA in tail ^#^]	3.70 ± 1.48	3.66 ±1.65	3.67 ± 0.97	3.75 ± 1.68	0.953
H_2_O_2_ [%DNA in tail ^#^]	10.67 ± 2.21	10.76± 2.28	10.03 ± 1.90	11.12 ± 2.30	0.067
FPG [%DNA in tail ^#^]	7.40 ± 1.72	7.21 ± 1.70	7.34 ± 1.49	7.64 ± 1.93	0.483
GSH [µmol/L]	16.61 ± 3.68	15.94 ± 3.54	17.23 ±3.66	16.77 ± 3.81	0.255
GSSG [µmol/L]	8.40 ± 1.38	8.36 ± 1.47	8.34 ± 1.32	8.50 ± 1.37	0.830
GSH:GSSG ratio [-]	1.99 ± 0.35	1.92 ± 0.34	2.07 ± 0.31	1.99 ± 0.38	0.132
Total-C [mmol/L]	11.37 ± 2.30	11.03 ± 2.33	11.20 ± 1.96	11.85 ± 2.50	0.197
LDL-C [mmol/L]	6.52 ± 2.07	6.27 ± 2.01	6.38 ± 1.77	6.89 ± 2.33	0.308
HDL-C [mmol/L]	3.57 ± 0.92	3.43 ± 1.07	3.65 ± 0.84	3.63 ± 0.83	0.462
TG [mmol/L]	6.36 ± 2.75	6.49 ± 2.76	5.83 ± 2.52	6.68 ± 2.91	0.328
TG-HDL-ratio	2.00 ± 1.20	2.17 ± 1.29	1.78 ± 1.11	2.01 ± 1.17	0.336
CRP [mg/L]	2.18 ± 2.40	1.55 ± 1.57 *	3.05 ± 3.09 *	2.08 ± 2.24	0.013

Values are shown as mean ± stdv. *p*-Values refer to differences between groups (one-way ANOVA, Chi Square Test). CON (control group = observation only); RP (recommended protein group); HP (high protein group); BMI (body mass index); GSH (reduced glutathione); GSSG (oxidized glutathione); C (cholesterol); TG (triglycerides); * different to CON (*p* < 0.05); ^#^ assessed in whole blood.

**Table 2:** Baseline characteristics and nutrient intake of the participants in the New Zealand study.

	Total	RDA	2RDA	*p*-Value
Age [years]	74.2 ± 3.6	74.7 ± 3.9	73.7 ± 3.3	0.342
Body mass [kg]	84.2 ± 15.6	85.3 ± 20.1	82.5 ± 7.9	0.320
Height [m]	1.72 ± 0.07	1.73 ± 0.08	1.72 ± 0.06	0.874
BMI [kg/m^2^]	28.2 ± 4.2	28.4 ± 5.1	28.2 ± 3.3	0.268
Energy intake [kcal/d]	2316 ± 523	2488 ± 460	2132 ± 539	0.068
Protein intake [g/kg BW/d]	1.23 ± 0.28	1.26 ± 0.27	1.20 ± 0.29	0.616
Protein intake [g/d]	102.1 ± 23.9	107.3 ± 26.4	96.6 ± 20.4	0.234
Fat intake [g/kg BW/d]	1.09 ± 0.34	1.17 ± 0.31	1.01 ± 0.37	0.218
Fat intake [g/d]	89.9 ± 25.8	99.1 ± 26.0	80.0 ± 22.3	0.043
Carbohydrate intake [g/kg BW/d]	3.00 ± 1.15	3.10 ± 0.92	2.89 ± 1.38	0.637
Carbohydrate intake [g/d]	243.3 ± 76.7	260.0 ± 62.0	225.4 ± 88.6	0.238
Lysis [%DNA in tail ^#^]	4.72 ± 3.21	4.37 ± 2.91	5.10 ± 3.58	0.547
H_2_O_2_ [%DNA in tail ^#^]	4.91 ± 2.39	4.73 ± 2.76	5.11 ± 2.00	0.677
FPG [%DNA in tail ^#^]	3.37 ± 1.95	7.20 ± 1.72	3.27 ± 2.31	0.600
GSH [µmol/L]	17.46 ± 3.63	18.13 ± 3.91	16.73 ± 3.30	0.308
GSSG [µmol/L]	6.51 ± 1.91	6.54 ± 2.30	6.49 ± 1.48	0.948
GSH:GSSG ratio	2.96 ± 1.30	3.11 ± 11.37	2.80 ± 1.23	0.528
Total-C [mmol/L]	4.64 ± 0.88	4.77 ± 1.04	4.52 ± 0.71	0.445
LDL-C [mmol/L]	2.95 ± 0.91	2.81 ± 0.76	3.09 ± 1.06	0.428
HDL-C [mmol/L]	1.34 ± 0.40	1.34 ±0.44	1.35 ± 0.37	0.941
TG [mmol/L]	1.13 ± 0.52	1.17 ± 0.39	1.08 ± 0.64	0.654
TG-HDL_ratio	0.97 ± 0.70	1.00 ± 0.51	0.94 ± 0.88	0.816
CRP [mg/L]	1.98 ± 2.49	1.63 ± 2.09	2.18 ± 2.92	0.564

Values are shown as mean ± stdv. *p*-Values refer to differences between groups (unpaired *t*-test). RDA (recommended protein group); 2RDA (high protein group); BMI (body mass index); GSH (reduced glutathione); GSSG (oxidized glutathione); C (cholesterol); TG (triglycerides); ^#^ assessed in PBMC.

**Table 3:** Intervention effects on macronutrient and energy intake in the Austrian study (*n* = 119).

Parameter	Group	Pre (t1)	Post (t2)	Time *p*-Value	Group *p*-Value	Time × Group *p*-Value
Protein [g/kg BW/d]	CON	0.83 ± 0.40	0.91 ± 0.36	<0.001	<0.001	<0.001
	RP	0.89 ± 0.28	1.08 ± 0.32 **			
	HP	0.79 ± 0.31	1.54 ± 0.35 ***			
Carbohydrates [g/kg BW/d]	CON	2.68 ± 1.38	2.70 ± 1.10	0.566	0.891	0.408
	RP	2.72 ± 1.11	2.83 ± 0.95			
	HP	2.46 ± 1.21	2.48 ± 0.88			
Fat [g/kg BW/d]	CON	1.08 ± 0.56	1.18 ± 0.44	0.100	0.699	0.876
	RP	1.08 ± 0.39	1.12 ± 0.36			
	HP	1.03 ± 0.52	1.09 ± 0.38			
Energy intake [kcal/d]	CON	1838 ± 726	1961 ± 578	0.001	0.475	0.332
	RP	1981 ± 690	2118 ± 630			
	HP	1730 ± 700	1989 ± 525 **			

Values are shown as mean ± stdv. *p*-Values refer to main effects of time and group as well as time*group interactions (two-way mixed ANOVA). Significant effects (*p* < 0.05) are shown in bold. In case of significant overall time effects, Bonferroni-corrected post hoc analyses were performed individually for groups, whereby asterisks indicate significant differences to pre (t1). *** (*p* < 0.001); ** (*p* < 0.01). CON (control group = observation only); RP (recommended protein group); HP (high protein group).

**Table 4:** Intervention effects on macronutrient and energy intake in the New Zealand study (*n* = 29).

Parameter	Group	Pre (t1)	Post (t2)	Time *p*-Value	Group *p*-Value	Time × Group *p*-Value
Protein [g/kg BW/d]	RDA	1.26 ± 0.27	0.89 ± 0.09 ***	0.164	<0.001	<0.001
	2RDA	1.20 ± 0.29	1.74 ± 0.22 ***			
Carbohydrates [g/kg BW/d]	RDA	3.10 ± 0.92	4.18 ± 0.83 **	<0.001	0.794	0.397
	2RDA	2.89 ± 1.38	4.21 ± 0.89 ***			
Fat [g/kg BW/d]	RDA	1.17 ± 0.31	1.11 ± 0.23	0.706	0.359	0.164
	2RDA	1.01 ± 0.37	1.12 ± 0.18			
Energy intake [kcal/d]	RDA	2488 ± 460	2683 ± 516	<0.001	0.423	0.009
	2RDA	2132 ± 539	2823 ± 153 ***			

Values are shown as mean ± stdv. *p*-values refer to main effects of time and group as well as time*group interactions(two-way mixed ANOVA). Significant effects (*p* < 0.05) are shown in bold. In case of significant overall time effects, Bonferroni-corrected post hoc analyses were performed individually for groups, whereby asterisks indicate significant differences to pre (t1). *** (*p* < 0.001); ** (*p* < 0.01). RDA (Recommended Daily Allowance for protein, 0.8 g/kg BW/d).

**Table 5:** Correlations of DNA damage markers with plasma lipid biomarkers at baseline in the Austrian study.

Biomarker	Lysis [%DNA in Tail]	H_2_O_2_ [%DNA in Tail]	FPG [%DNA in Tail]
Total-C [mg/dL]	0.472 **	0.256 **	0.394 **
HDL-C [mg/dL]	−0.243 **	−0.169	−0.027
LDL-C [mg/dL]	0.462 **	0.265 **	0.377 **
TG [mg/dL]	0.615 **	0.365 **	0.281 **
Total-C/HDL-C	0.600 **	0.364 **	0.296 **

C (cholesterol); TG (triglycerides); ** *p* < 0.01.

**Table 6:** Impact of the protein intervention on DNA damage marker in the Austrian study.

Parameter	Group	Mean ± Stdv		Time	Group	Time × Group	Time Points Differences
		Baseline	6 Weeks	*p*-Value	*p*-Value	*p*-Value	Δ (Post–Pre)
Lysis [%DNA in tail]	CON	3.74 ± 1.73	3.28 ± 1.19 ***	0.002	0.446	0.263	−0.45 ± 1.24
	RP	3.64 ± 0.99	3.54 ± 1.32				−0.10 ± 1.51
	HP	3.55 ± 1.17	3.03 ± 0.84 ***				−0.53 ± 0.82
H_2_O_2_ [%DNA in tail]	CON	10.92 ± 2.35	9.69 ± 2.06 ***	<0.001	0.363	0.156	−1.23 ± 1.83
	RP	10.00 ± 1.89	9.43 ± 1.92				−0.58 ± 1.96
	HP	10.73 ± 2.02	9.40 ± 1.94 ***				−1.33 ± 1.74
FPG [%DNA in tail]	CON	7.23 ± 1.79	6.33 ± 1.51 ***	<0.001	0.698	0.148	−1.15 ± 1.61
	RP	7.32 ± 1.49	6.78 ± 1.91 **				−0.54 ± 1.96
	HP	7.45 ± 1.93	6.18 ± 1.64 ***				−1.54 ± 1.67
GSH [µmol/L]	CON	15.98 ± 3.71	15.58 ± 2.69	0.002	0.373	0.417	−0.40 ± 2.91
	RP	17.34 ± 3.69	16.10 ± 2.91 *				−1.24 ± 2.86
	HP	16.74 ± 3.93	15.96 ± 2.35 *				−0.77 ± 2.54
GSSG [µmol/L]	CON	8.23 ± 1.47	8.17 ± 1.46	0.172	0.831	0.764	−0.06 ± 1.61
	RP	8.40 ± 1.35	8.21 ± 1.49				−0.20 ± 1.29
	HP	8.51 ± 1.45	8.22 ± 1.09				−0.29 ± 1.37
GSH:GSSG ratio	CON	1.95 ± 0.34	1.95 ± 0.37	0.302	0.440	0.727	−0.001 ± 0.43
	RP	2.07 ± 0.31	2.00 ± 0.39				−0.07 ± 0.33
	HP	1.99 ± 0.39	1.96 ± 0.28				−0.03 ± 0.28
CRP [mg/L]	CON	1.48 ± 1.47	2.62 ± 6.79	0.207	0.459	0.385	1.15 ± 6.71
	RP	3.10 ± 3.21	2.76 ± 2.67				−0.34 ± 3.30
	HP	2.05 ± 2.31	3.03 ± 5.09				0.99 ± 4.64

Values are shown as mean ± stdv. *p*-Values refer to main effects of time, group and time*group interactions (two-way mixed ANOVA). Significant effects are shown in bold. In case of significant overall time effects, Bonferroni-corrected post hoc analyses were performed individually for groups, whereby asterisks indicate significant differences to pre (t1). *** (*p* < 0.001); ** (*p* < 0.01); * (*p* < 0.05). CON (control group = observation only); RP (recommended protein group); HP (high protein group).

**Table 7:** Impact of the protein intervention on DNA damage marker in the New Zealand study.

Parameter	Group	Mean ± Stdv		Time	Group	Time × Group	Time Points Differences
		Baseline	10 Weeks	*p*-Value	*p*-Value	*p*-Value	Δ (post–pre)
Lysis [%DNA in tail]	RDA	5.10 ± 3.58	4.62 ± 3.16	0.72	0.729	0.348	0.22 ± 1.68
	2RDA	4.37 ± 2.91	4.58 ± 2.94				−0.48 ± 2.22
H_2_O_2_ [%DNA in tail]	RDA	5.11± 2.00	4.90 ± 2.71	0.608	0.643	0.999	−0.21 ± 2.06
	2RDA	4.73 ± 2.76	4.52 ± 2.14				−0.21 ± 2.26
FPG [%DNA in tail]	RDA	3.27 ± 2.31	3.36 ± 1.81	0.869	0.614	0.994	0.09 ± 2.68
	2RDA	3.46 ± 1.64	3.54 ± 2.40				0.09 ± 3.10
GSH [µmol/L]	RDA	16.73 ± 3.30	16.37 ± 1.26	0.059	0.475	0.189	−1.24 ± 2.86
	2RDA	18.13 ± 3.91	16.24 ± 1.87				−0.72 ± 2.54
GSSG [µmol/L]	RDA	6.49 ± 1.48	6.87 ± 1.72	0.657	0.796	0.518	−0.07 ± 2.02
	2RDA	6.57 ± 2.30	6.47 ± 2.43				0.38 ± 1.61
GSH:GSSG ratio	RDA	2.80 ± 1.23	2.66 ± 1.34	0.146	0.806	0.545	−0.32 ± 1.05
	2RDA	3.11 ± 1.38	2.78 ± 0.87				−0.14 ± 0.50
CRP [mg/L]	RDA	2.18± 2.92	2.87 ± 3.34	0.71	0.281	0.28	−0.34 ± 2.63
	2RDA	1.74 ± 2.23	1.39 ± 1.08				0.70 ± 2.27

Values are shown as mean ± stdv. *p*-Values refer to main effects of time and time*group interactions (two-way mixed ANOVA). Significant effects are shown in bold. RDA (recommended protein intake); 2RDA (twofold recommended dietary intake).

## Data Availability

The data presented in this study are available in this article.

## Supplementary Materials

The following are available online at https://www.mdpi.com/article/10.3390/nu13103479/s1, Table S1: Correlations of DNA damage markers with plasma lipid parameters at baseline in males and females from the Austrian study; Table S2: Impact of the protein intervention on DNA damage markers in females from the Austrian study. Table S3: Impact of the protein intervention on DNA damage marker in males from the Austrian study.
