# Supplementary material for: The Effect of Elevated Protein Intake on DNA Damage in Older People: Comparative Secondary Analysis of Two Randomized Controlled Trials
Source: Nutrients. 2021 Sep 30;13(10):3479. doi: 10.3390/nu13103479 (PMC8537980; doi:10.3390/nu13103479)
Supplement: Supplementary file 1 [file nutrients-13-03479-s001.zip › nutrients-1365200-supplementary.pdf]

Supplementary Materials

Supplementary Table S1. Correlations of DNA damage markers with plasma lipid parameters at baseline in males and females from the Austrian study.

	Females			Males		
	Lysis [%DNA in tail]	H2O2 %DNA in tail]	FPG [%DNA in tail]	Lysis [%DNA in tail]	H2O2 %DNA in tail]	FPG [%DNA in tail]
Total-cholesterol [mg/dl]	**0.590****	**0.284***	**0.429****	**0.311***	0.123	**0.356****
HDL-cholesterol [mg/dl]	**-0.330****	**-0.332****	-0.185	**-0.256***	-0.143	0.128
LDL-cholesterol [mg/dl]	**0.544****	**0.296***	**0.389****	**0.316***	0.147	**0.348****
Triglyceride [mg/dl]	**0.688****	**0.484****	**0.442****	**0.499****	0.201	0.044
TG/HDL [-]	**0.672****	**0.473****	**0.393****	**0.498****	0.232	0.163
Chol/HDL [-]	**0.688****	**0.503****	**0.411****	**0.478****	0.189	-0.064

* p<0.05, ** p<0.01

Supplementary Table S2. Impact of the protein intervention on DNA damage markers in females from the Austrian study.

Parameter	Group	Mean ± stdv		time	group	time x group	Time points differences
		Baseline	6 weeks	p-value	p-value	p-value	Δ (post – pre)
**Lysis [%DNA in tail]**	CON	4.05±2.14	3.28±1.43*				-0.77±1.41
	RP	3.70±1.17	3.59±1.39	**0.011**	0.451	0.294	-0.11±1.65
	HP	3.47±0.93	3.02±0.86*				-0.47±0.86
**H_2O_2 [%DNA in tail]**	CON	11.33±2.74	9.60±2.21***				-1.74±1.48
	RP	10.04±1.99	9.07±2.19	**< 0.001**	0.319	0.396	-0.97±2.22
	HP	10.91±1.75	9.67±2.16**				-1.44±1.58
**FPG [%DNA in tail]**	CON	7.35±2.08	6.56±1.48*				-0.79±1.44
	RP	7.50±1.63	6.25±1.63**	**< 0.001**	0.862	0.608	-1.26±1.80
	HP	7.32±1.75	6.10±1.80**				-1.11±1.79
**GSH [µmol/l]**	CON	15.08±3.35	14.81±2.07				-0.27±3.19
	RP	17.62±3.74	15.82±3.34*	**0.028**	0.101	0.196	-1.79±2.86
	HP	16.33±3.32	15.94±1.95				-0.39±2.49
**GSSG [µmol/l]**	CON	8.06±1.44	7.78±1.69				-0.28±1.77
	RP	8.12±1.49	7.56±1.68*	0.131	0.651	0.545	-0.56±1.09
	HP	8.20±1.32	8.17±1.10				-0.04±1.48
**GSH:GSSG ratio**	CON	1.88±0.30	1.97±0.42				0.09±0.38
	RP	2.17±0.27	2.15±0.47	0.839	0.061	0.428	-0.03±0.41
	HP	2.02±0.38	1.98±0.28				-0.04±0.29
**CRP [mg/l]**	CON	1.74±1.88	3.60±9.23				1.86±9.18
	RP	2.63±2.03	3.08±2.79	0.314	0.919	0.496	0.46±2.38
	HP	2.50±2.60	2.36±1.96				-0.13±1.72

Values are shown as mean±stdv. p-Values refer to main effects of time, group and time*group interactions (two-way mixed ANOVA). Significant effects are shown in bold. In case of significant overall time effects, Bonferroni-corrected post hoc analyses were performed individually for groups, whereby asterisks indicate

significant differences to pre (t1). *** (p<0.001); ** (p<0.01); * (p<0.05). CON (control group = observation only); RP (recommended protein group); HP (high protein group).

Supplementary Table S3. Impact of the protein intervention on DNA damage marker in males from the Austrian study.

Parameter	Group	Mean ± stdv		time	group	time x group	Time points differences
		Baseline	6 weeks	p-value	p-value	p-value	Δ (post – pre)
**Lysis [%DNA in tail]**	CON	3.37±1.01	3.28±0.87				-0.12±0.91
	RP	3.59±0.78	3.49±1.28	0.066	0.746	0.222	-0.09±1.39
	HP	3.66±1.42	3.04±0.85**				-0.61±0.81
**H_2O_2 [%DNA in tail]**	CON	10.44±1.75	9.79±1.93				-0.69±2.10
	RP	9.97±1.84	9.80±1.55	**0.003**	0.825	0.109	-0.16±1.59
	HP	10.51±2.33	9.09±1.65**				-1.42±1.71
**FPG [%DNA in tail]**	CON	7.09±1.43	6.07±1.55**				-0.94±1.50
	RP	7.14±1.36	7.35±2.06	**0.001**	0.431	**0.006**	0.21±1.64
	HP	7.59±2.16	6.27±1.48***				-1.32±1.20
**GSH [µmol/l]**	CON	17.01±3.91	16.47±3.09				-0.55±2.63
	RP	17.06±3.73	16.37±2.48	**0.027**	0.990	0.723	-0.69±2.84
	HP	17.20±4.58	15.99±2.79				-1.22±2.60
**GSSG [µmol/l]**	CON	8.43±1.53	8.63±1.01				0.19±1.39
	RP	8.69±1.17	8.85±0.94	0.686	0.760	0.139	0.17±1.39
	HP	8.86±1.55	8.28±1.10				-0.58±1.20
**GSH:GSSG ratio**	CON	2.03±0.37	1.91±0.31				-0.11±0.47
	RP	1.96±0.31	1.85±0.23	0.080	0.792	0.639	-0.11±0.23
	HP	1.96±0.42	1.94±0.29				-0.02±0.28
**CRP [mg/l]**	CON	1.17±0.71	1.49±0.74*				0.32±0.61
	RP	3.61±4.11	2.43±2.56	0.421	0.185	0.063	-1.18±3.95
	HP	1.53±1.85	3.81±7.20				2.28±6.41

Values are shown as mean±stdv. p-Values refer to main effects of time, group and time*group interactions (two-way mixed ANOVA). Significant effects are shown in bold. In case of significant overall time effects, Bonferroni-corrected post hoc analyses were performed individually for groups, whereby asterisks indicate significant differences to pre (t1). *** (p<0.001); ** (p<0.01); * (p<0.05). CON (control group = observation only); RP (recommended protein group); HP (high protein group).
